# Exploring Association of Breast Pain, Pregnancy, and Body Mass Index with Breast Tissue Elasticity in Healthy Women: Glandular and Fat Differences

**DOI:** 10.3390/diagnostics10060393

**Published:** 2020-06-10

**Authors:** Martina Dzoic Dominkovic, Gordana Ivanac, Kristina Bojanic, Kristina Kralik, Martina Smolic, Eugen Divjak, Robert Smolic, Boris Brkljacic

**Affiliations:** 1 Department of Radiology, General Hospital Orasje, 3rd Street, 76270 Orasje, Bosnia and Herzegovina; martina.dzoic@gmail.com; 2 Department of Diagnostic and Interventional Radiology, University Hospital Dubrava, Avenue Gojka Suska 6, 10000 Zagreb, Croatia; gordana.augustan@gmail.com (G.I.); edivjak@gmail.com (E.D.); boris@brkljacic.com (B.B.); 3 University of Zagreb School of Medicine, Salata 3, 10000 Zagreb, Croatia; 4 Department of Biophysics and Radiology, Faculty of Dental Medicine and Health Osijek, J.J. Strossmayer University of Osijek, 31000 Osijek, Croatia; kristina.bojanic@dzo.hr; 5 Department of Biophysics and Radiology, Faculty of Medicine Osijek, J.J. Strossmayer University of Osijek, 31000 Osijek, Croatia; 6 Department of Radiology, Health Center Osijek, 31000 Osijek, Croatia; 7 Department of Medical Statistics and Medical Informatics, Faculty of Medicine Osijek, J.J. Strossmayer University of Osijek, 31000 Osijek, Croatia; kristina.kralik@mefos.hr; 8 Department of Pharmacology and Biochemistry, Faculty of Dental Medicine and Health Osijek, J.J. Strossmayer University of Osijek, 31000 Osijek, Croatia; martina.smolic@mefos.hr; 9 Department of Pharmacology, Faculty of Medicine Osijek, J.J. Strossmayer University of Osijek, 31000 Osijek, Croatia; 10 Department of Pathophysiology, Physiology and Immunology, Faculty of Dental Medicine and Health Osijek, J.J. Strossmayer University of Osijek, 31000 Osijek, Croatia; 11 Department of Pathophysiology, Faculty of Medicine Osijek, J.J. Strossmayer University of Osijek, 31000 Osijek, Croatia; 12 Department of Medicine, Division of Gastroenterology/Hepatology, University Hospital Osijek, 31000 Osijek, Croatia

**Keywords:** breast, ultrasonography, shear wave elastography, mastodynia, body mass index

## Abstract

Breast sonoelastography is a relatively novel ultrasound (US) method that enables estimation of tissue stiffness to estimate the elasticity of normal breast tissue and seek to correlate it with well-known breast cancer risk factors. Two hundred women of different age were included in the study and completed a questionnaire about personal, familiar, and reproductive history. Glandular and fatty tissue elasticity in all breast quadrants was measured by shear wave elastography (SWE). Mean elastographic values of breast tissue were calculated and compared to personal history risk factors. Elasticity of normal glandular tissue (66.4 kilopascals (kPa)) was higher than fatty tissue (26.1 kPa) in all breast quadrants and in both breasts. Lower outer quadrant (LOQ) had the lowest elasticity values of both parenchyma and fat. Higher elasticity values of breast tissue were confirmed in the left breast than in the right breast. Glandular and fat tissue elasticity negatively correlated with body mass index (BMI). Women with mastodynia had higher glandular elastographic values compared to subjects without breast pain. Nuliparity was also associated with higher elasticity of glandular breast tissue. The results of this study are promising and could, over time, contribute to a better understanding of glandular breast tissue elasticity as a potential risk factor for breast cancer.

## 1. Introduction

Younger women typically have breasts with higher percent of glandular tissue (denser breasts). Breasts are exposed to hormonal changes during menstrual cycle [1,2,3]. They reach full development during pregnancy and lactation [3]. In addition, there is post-lactation phase with remodeling of breast tissue and a significant number of breast cell death. After every pregnancy, there is a reduction in breast parenchyma quantity and an increase in quantity of fat and connective tissue. Involution of breast parenchyma starts in the postmenopausal phase, and it is replaced with fat tissue, thus making the breast become less dense. Additionally, ligaments lose their elasticity, causing relaxation of breasts [1,2,3].

The main risk factors for breast cancer are: advanced age, early menarche and late menopause, nulliparity, older age at first pregnancy (more than 30 years), personal history of breast disease (especially epithelial hyperplasia with and without atypia, carcinoma in situ), lifestyle (alcohol consumption and lack of folic acid, smoking), use of exogenous hormones, and a family history of breast cancer [4].

Evaluation of stiffness of different breast lesions with sonoelastography was widely studied over the last years [5]. Two main techniques are in use, strain and shear wave elastography (SWE), the latter providing a quantitative and therefore objective assessment of breast tissue elasticity. SWE transmits shear waves in tissue and measures speed of its propagation through the tissue. Speed of shear wave propagation is proportional to tissue elasticity [6].

The value of sonoelastography in differentiation of benign and malignant breast lesions and cut off values between these lesions are well-documented [6,7,8,9,10,11,12].

However, there is a lack of studies determining elasticity of healthy breast tissue. Correlations of breast elasticity with medical history data have been evaluated only by few studies [13,14]. In addition, data in recent literature are sparse on the association of known breast cancer risk factors with breast tissue elasticity. In contrast, it is well known how the main breast cancer risk factors correlate with mammographic breast density, which alone is itself also a breast cancer risk factor [15]. Increasing age and pregnancies showed negative correlation with breast mammographic density due to water breast content [13]. The connection of breast tissue elasticity and certain physiologic factors (such as lactation, hormonal status, menstrual cycle, age, and others) could be a new promising research area, for further clinical applications, currently without a straightforward answer.

Moreover, an interesting question, still unanswered, is how glandular breast tissue elasticity corresponds to breast fibroglandular density. Elasticity could also be a potential breast cancer risk factor, like breast density on mammography [13].

Therefore, the aim of our study was to estimate the elasticity of normal breast tissue and to establish a possible correlation of it with the well known breast cancer risk factors.

## 2. Participants and Methods

### 2.1. Participants

The study was reviewed and approved by the University of Zagreb, School of Medicine Ethical Committee on 23 February 2017 (Approval No. 641-01/17-02). Explanation of informed consent was provided by researchers to all potential participants meeting inclusion criteria, and, afterwards, participants willing to be included in the study signed an informed consent form before being included in the study. Subjects were included successively if they agreed to participate in the study and did not meet the exclusion criteria. During a one-year period, we included a total of two hundred women in the study. Study participants consisted of healthy volunteers (that is, without being diagnosed with breast disease), asymptomatic women that underwent mammography screening according to national guidelines, and those who additionally underwent sonoelastography. Women with symptoms that were examined due to breast pain or breast palpable mass were also included in the study. Women with any prior breast surgery, prior thoracic radiation, and women with known breast tumors were excluded from the study.

A self-reported questionnaire was used to investigate any potential associations between breast elasticity and known breast cancer risk factors, such as age, age of first menstruation, current phase of menstrual cycle (day of menstrual cycle for premenopausal women or age of menopause for postmenopausal women), number of pregnancies, age at first pregnancy, duration of lactation, family history of breast cancer, presence of breast pain related to menstrual cycle in the luteal phase, body mass index, exogenous hormones (oral contraception or hormonal replacement therapy), and consumption of alcohol and nicotine.

### 2.2. Elastography and Analysis

All patients underwent SWE examination on an Aixplorer scanner (Supersonic Imagine, Aix en Provance, France, software version 6.2.23751, product version 6.2.0) with a high-frequency 4–15 MHz linear transducer. The complete diagnostic procedure was performed by one well-experienced breast radiologist with >5 years of experience in breast radiology so that there was no “interobserver” variability. First, classical gray-scale (B-mode) ultrasound (US) examination was performed.

**Figure 1:**
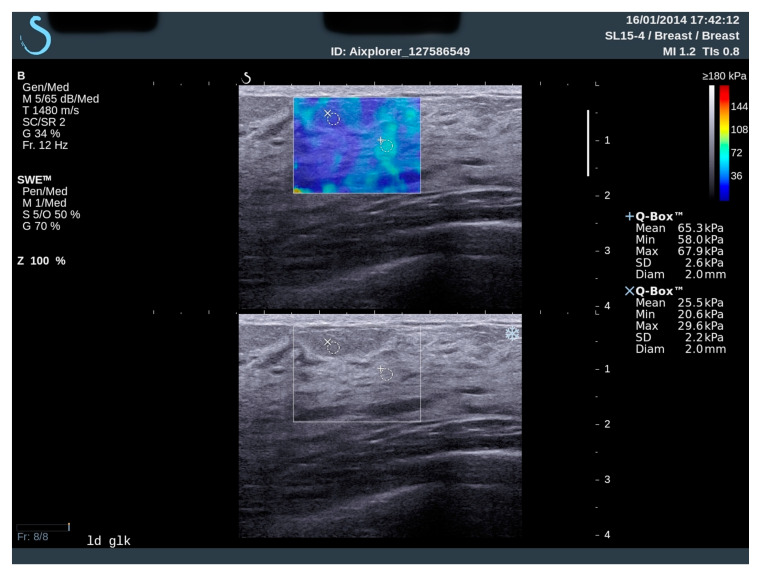
Elastography measurement. Region of interest is placed into representative place for fatty and glandular breast tissue. Ultrasound device shows elasticity values in color and kPa.

After B-mode examination, the SWE was performed, without manual compression. On every elastographic image, tissue elasticity was presented in color, from dark blue lesions with stiffness just above 0 kPa or 0 m/s to red color that represents high stiffness; for our study, the highest values were placed on >180 kPa (7.7 m/s) (Figure 1).

The stiffness of the tissue was measured by the built-in quantification region of interest (Q-Box; SuperSonic Imagine) of the system. All elastographic measurements were obtained with a 2-mm Region of Interest (ROI) that was placed in epithelial (parenchyma) and fat breast tissue, according to B-mod examination. In every elastographic measurement, subcutaneous fat tissue at the top of the screen and pectoral muscle at the bottom of the screen was present. Every breast was divided in four quadrants, and a single measurement per quadrant was performed. All measurements were performed using the same “breast” preset of the ultrasound scanner.

Mean, minimal, and maximum elastographic value in Q box were measured. Eight measurements were performed, 4 in each breast on every participant. Altogether, 48 different values for every woman (breast parenchyma and fat tissue) were obtained. The mean elastographic values of breast parenchyma and fat tissue were used for further processing, so 16 elastographic values per woman, one mean value for parenchyma and one mean value for fat tissue per each breast quadrant in both breasts, were used for further evaluations.

All pictures were stored in the digital format. All data were stored in Microsoft Excel sheet (Microsoft Corporation, Redmond, WA, USA).

### 2.3. Statistical Analysis

Data were described using descriptive statistical methods. Categorical values were presented as absolute and relative frequencies, and numeric variables as median and interquartile range in case of deviation from normal distribution. Shapiro-Wilk’s test was used to analyze distribution of numeric variables and according to these findings, non-parametric tests were used. The Mann–Whitney *U* test was used for comparison differences between two independent groups. The Friedman’s test and Wilcoxon Signed–Rank Test for Paired Samples were used for comparison differences between numeric variables in case of two or more dependent groups. The Pearson’s *r* test was used to determine the association between normally distributed variables. Stepwise multiple regression analysis was used to determine the predictors affecting elastography values of glandular breast tissue. All *p* values were two-sided. The level of significance was set to Alpha = 0.05. The statistical analysis was performed using MedCalc Statistical Software version 19.1.7 (MedCalc Software Ltd., Ostend, Belgium; https://www.medcalc.org; 2020) and IBM SPSS Statistics 23 (IBM Corp., released 2015; IBM SPSS Statistics for Windows, Version 23.0. Armonk, NY, USA: IBM Corp.)

## 3. Results

Socio-demographic and clinical characteristics of subjects involved in the study are presented in Table 1.

The range of age was 17 to 73 years, with median age of 37 (29–52). (Table 2). Descriptive statistics of quantitative socio-demographic and clinical variables are presented in the Table 2.

Median values of mean elastographic values of breast parenchyma, 66.4 kPa (48.1–86.7), were significantly higher than values of fatty tissue 26.1 kPa (21.1–32.5) (chi-squared Friedman’s test, *p* ˂ 0.05). This was also determined for median values of minimal and maximal elastographic values.

In all breast quadrants, elastographic values of glandular tissue were significantly higher compared to fat tissue (Mann–Whitney *U* test, *p* < 0.001). A statistically significant difference in the elasticity of the glandular parenchyma was determined for the lower outer quadrant (LOQ) compared to all other quadrants. The glandular tissue elasticity in the upper lateral quadrant (UOQ) was statistically significantly higher compared to LOQ and to lower inner quadrant (LIQ) (Friedman’s test, Post hoc Conover, *p* < 0.001). Median values of stiffness in all quadrants are presented in Table 3.

Statistically higher elastographic values were observed in the left breast for both glandular and fat tissue (Table 4).

The differences in the elastographic values of glandular and fatty breast tissue in relation to certain anamnestic data are shown in Table 5. Statistically significant difference between the glandular elasticity values and breast pain (Mann–Whitney *U* test, *p* = 0.02) was obtained. Higher elastographic value of the glandular parenchyma (72.2 kPa (51.8–90.8)) was determined in subjects with breast pain in regards to subjects without pain, with glandular breast tissue elasticity of 61.7 kPa (42.2–83.5). Women who had ever been pregnant had significantly lower glandular elastographic values (61.7 (42.2–80.03)) compared to women who had never been pregnant (72.2 (52.8–93.6)) (Mann–Whitney *U* test, *p* = 0.001). No statistically significant differences in the elasticity of the glandular parenchyma with respect to certain other examined parameters (family history of breast cancer, taking oral contraceptives (OC) or hormone replacement therapy (HRT), and smoking and alcohol consumption) were detected.

The differences in elastographic values of fatty tissue in regard to examined anamnestic data were not statistically significant.

Higher elastographic values of glandular breast parenchyma, although not statistically significant, were noted in younger women, women with earlier menarche, earlier menopause, women in the first phase of cycle at the time of the examination, women who were older in first pregnancy, and women with shorter lactation duration.

**Figure 2:**
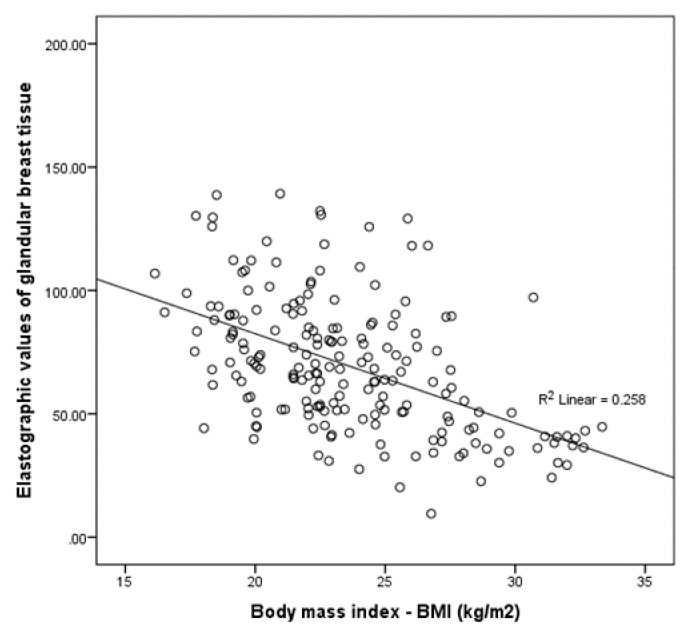
Pearson’s coefficient correlation (*r*) between elastographic values of glandular breast tissue and body mass index (BMI) (kg/m^2^) (*r*= −0.508; *p* < 0.001; 95% Confidence Interval (CI) for *r* −0.606 to −0.395).

Since outliers were detected in 5 subjects with respect to body mass index (BMI), these 5 subjects were excluded from correlation calculation. Elastographic values of glandular breast tissue demonstrated negative correlation with BMI (Pearson’s coefficient correlation (*r*) between elastographic values of glandular breast tissue and body mass index (BMI) (kg/m^2^) (*r* = −0.508; *p* < 0.001; 95% Confidence Interval (CI) for *r* −0.606 to −0.395 (Figure 2).

Negative correlation was also observed between elastographic values of breast fat tissue and BMI (Pearson’s coefficient correlation *r* = −0.268; *p* = 0.001; 95% CI for *r* −0.392 to −0.134). Correlation with other examined parameters (age, age of menarche, age of menopause, day of the menstrual cycle at the time of the examination, age of first pregnancy, duration of lactation, and number of lesions in the breast) did not reach statistical significance.

A weak correlation was observed between BMI and elastographic values of glandular breast tissue based on Stepwise multiple regression analysis (R^2^ = 0.246; β = −3.2, rparc = −0.496, rsemoparcial = 0.496, F = 64.2; *p* < 0.001).

## 4. Discussion

Features of different benign and malignant breast lesions were examined in recent studies by elastography [7,8,9,10,11,14]. However, there are very few studies in which normal breast tissue was analyzed, with very diverse results reported [14,16,17,18]. Here, we report of elastographic values of healthy breast tissue with average elastographic values of breast tissue of 66.4 kPa for glandular tissue and 26.1 kPa for fat tissue.

A study from Krouskup et al. reported elastographic values of breast tissue that were quite similar to our results (from 28 kPa to 66 kPa) [19]. Anthanasiou et al. reported values of 7 kPa for fat and 30–50 kPa for glandular tissue [14]. Rzymski et al. reported elastographic values of breast parenchyma 11.3 ± 5.8 kPa and of fat tissue 9.2 ± 4.5 measured in 101 women [13]. Young’s module varies from 3 kPa for fat tissue to 45 kPa for glandular tissue according to the study from Tanter et al. from 2008 [7]. Quite different results could be explained due to consequence of the different ways of performing measurements. Device settings are not always explained in detail in the Material and Methods sections of studies, and different “presets” and “modes” of device can influence measurements of elastographic parameters. Connective tissue and Cooper’s ligaments are interposed between fat lobes and serve as a support to glandular tissue of the breast, so they also contribute to the measured values in kPa. Currently, there is no technique that could exactly measure the level of the compression with the ultrasound probe and exclude “interobserver variability” [14]. Some authors suggested a way to determine the level of probe compression with variability of ≤10%; however, this technique has not been accepted in clinical setting because this would significantly prolong examination duration [18]. Magnetic resonance elastography (MRE) showed lower elasticity values of breast tissue (7.6 ± 3.6 kPa for breast parenchyma and 3.3 ± 1.9 kPa for fat tissue) [20,21]. Different Young’s modules in MR and US elastography could be the reason for the differences between MRE and sonoelastography: “supersonic shear” waves have frequencies of 250 Hz (frequency width 50–450 Hz), and frequencies used in MRE are 50–80 Hz [7].

The largest number of breast tumors arise from the UOQ. It is explained with the fact that the largest volume of breast parenchyma is located in that quadrant but also outer breast quadrants have higher genetic instability compared to the inner quadrants [22]. The highest stiffness in UOQ could also be one of causes for the fact that most tumors arise from this quadrant. But inner breast quadrants also show high elastographic values of breast parenchyma. A previous study showed higher elastographic values in inner quadrants compared to the outer quadrants [13]. This result could be explained with the fact that tumors arising in inner quadrants have worse outcomes [22] because of their higher histological grade compared with tumors arising from outer breast quadrants [23]. 

Our results showed higher elastographic values of glandular and fatty tissue in the left breast as compared to the right breast. One of possible explanations for that finding could be found in a well-known fact that breast cancers more often arise from the left breast [24]. In addition, a larger milk production during lactation in the right breast was determined earlier, and that might be protective factor for the breast cancer occurrence in the right breast [25].

Our research established the existence of differences in the elasticity of glandular and fat breast tissue with regard to certain risk and protective factors for breast cancer. Higher elastographic values of glandular breast tissue were observed in women with breast pain and in women who had never been pregnant. Regression analysis showed a weak correlation between BMI and the elasticity of the glandular parenchyma. However, research on a larger sample of women, along with the analysis of other potential confounding factors, could contribute to clarification of the obtained results. 

Higher elastographic values of glandular breast parenchyma, although without statistically significant differences, were noted in younger women, women with earlier menarche, earlier menopause, women in the first phase of cycle at the time of the examination, women who were older in first pregnancy, and women with shorter lactation duration. Lactation is a protective factor for breast cancer, and older age at first pregnancy and earlier menarche are risk factors for breast cancer, and these findings were expected. We hypothesized that women with later menopause would have higher elastographic values; however, this was not observed, but since statistical significance of observed differences were not reached, further studies are therefore needed. Rzymski et al. observed the positive correlation between elastographic values of glandular tissue and age of patients, as well as between elastographic values of fat tissue and duration of lactation [13]. Exogenous hormones, age, and pregnancy play an important role in histological breast changes, such as proliferation and apoptosis of breast parenchyma, as well as variations in breast volume and water content. Pregnancy causes hyperplasia of epithelial cells and a decrease in the amount of connective tissue. After lactation, the situation is opposite: epithelial cells disappear, and there is hyperplasia of connective tissue [13].

Women that reported mastalgia mostly during luteal phase of menstrual cycle had higher elastographic measurements. There are histological differences in breast tissue between follicular and luteal phase of menstrual cycle due to hormonal changes in the body of the woman [26,27]. In addition, there is a small increase in breast density during the second phase of the menstrual cycle, but it is not significant and does not decrease the sensitivity of mammography in luteal phase [27,28,29,30]. Wojcinski measured elastographic values of breast tissue during the menstrual cycle in women with and without oral contraceptives (OC). Women without OC had a continuous increase of breast parenchyma stiffness during their menstrual cycle, with the highest elastographic values in the luteal phase of menstrual cycle [31]. Breast changes in the menstrual cycle causing breast pain are accompanied with higher elastographic values of glandular breast tissue, implying that there are some changes in breast structure, volume, and the amount of the epithelial cells.

Limitation of our study lies in the fact that there was no standardization of sonoelastographic measurement that could decrease the subjectivity of the method and also influence on compression during examination. Other limitation is that our participants were not consisted of women of one age group or the particular phase of menstrual cycle; however, this was a first research from our group with the aim to elucidate major differences. Further studies are needed to explore the relevance of elasticity as an independent risk factor for breast cancer similar to the breast density or the hypothesis that different breast cancer risk factors can influence the breast tissue elasticity. We emphasize that our study of the elasticity of glandular and fatty breast tissue, given the very scarce data in the literature, can contribute to setting standards, as well as widening of clinical indications of elastography, e.g., radiotherapy or chemotherapy progress, rehabilitation, and complication after BC treatment and many others.

## 5. Conclusions

In conclusion, elasticity of normal breast parenchyma was higher than fatty tissue, with the lowest value in LOQ. The left breast had higher elasticity values of breast tissue compared to the right breast. Glandular and fatty tissue elasticity negatively correlated with BMI. Women with mastodynia had higher glandular elastographic values compared to subjects without breast pain. Nuliparity was also associated with higher elasticity of glandular breast tissue. The results of our research are promising and could, over time, contribute to a better understanding of glandular breast tissue elasticity as a potential risk factor for breast cancer.

## Figures and Tables

**Table 1:** Socio-demographic and clinical characteristics of participants involved in the study.

Subjects Characteristics		Total Number	%
Family history of breast cancer	Negative	166	83%
	Positive	34	17%
Exogenous hormones	No	146	73%
	Yes	54	27%
Smoking	No	131	65.5%
	Yes	69	34.5%
Alcohol consumption	No	135	67.5%
	Yes	65	32.5%
Breast pain	No	112	56%
	Yes	88	44%

**Table 2:** Descriptive statistics of quantitative socio-demographic and clinical variables.

Subjects Characteristics	
Age (years) *	37 (29–52)
Age of menarche *	13 (12–14)
Age of menopause *	50 (45–52)
Day of menstrual cycle at the time of the examination *	15 (8–20)
Women ever being pregnant [*n* (%)]	110 (55)
Age of first pregnancy *	26 (21–28)
Duration of lactation (months) *	8 (4–22)
Body mass index (kg/m^2^) *	23 (21–26.2)
Total number of breast lesions *	2 (1–3)
Number of malignant lesions [*n* (%)]	8 (4)
Number of benign lesions [*n* (%)]	94 (47)

* value displayed as median with interquartile range.

**Table 3:** Comparison of elastographic values of glandular and fat tissue in different breast quadrants.

Breast Quadrants	Elastographic Values of Glandular Breast Tissue		Elastographic Values of Fat Tissue in Breast		*p* ^§^
	Median (IQR)	*p* *	Median (IQR)	*p* *	
UOQ	69.6 (50.4–93.9)	<0.001 ^†^	26.5 (20.1–36.7)	<0.001 ^†^	<0.001
LOQ	60.3 (39.8–75.9)		21.18 (16.5–28.4)		<0.001
UIQ	69.5 (47.3–97.4)		28.5 (21.2–37.6)		<0.001
LIQ	65.5 (43.9–94.1)		25.28 (19.1–34.3)		<0.001

UOQ—upper outer quadrant; LOQ—lower outer quadrant; UIQ—upper inner quadrant; LIQ—lower inner quadrant; IQR—interquartile range; * Friedman’s test (Post hoc Conover); ^§^ Mann–Whitney *U* test; ^†^ at *p* < 0.05 level significant differences between UOQ vs. LOQ. UOQ vs. LIQ. LOQ vs. UIQ. LOQ vs. LIQ.

**Table 4:** Comparison of elastographic values of glandular and fat tissue in left and right breast.

Breast	Elastographic Values of Glandular Breast Tissue		Elastographic Values of Fat Tissue in Breast		*p* ^†^
	Median (IQR)	*p* *	Median (IQR)	*p* *	
Left	68.9 (51.2–95.4)	0.005	27.1 (26–28.4)	<0.001	<0.001
Right	63.3 (47.7–85.3)		24.3 (18.4–31.7)		<0.001

IQR—interquartile range; * Wilcoxon Signed-Rank Test for Paired Samples; ^†^ Mann–Whitney *U* test.

**Table 5:** Elastographic values of glandular and fat tissue to data obtained from medical history.

Medical History Data		Elastographic Values of Glandular Breast Tissue		Elastographic Values of Fat Tissue in Breast	
		Median (IQR)	*p* *	Median (IQR)	*p* *
Family history of BC	no	65.2 (49.5–83.6)	0.25	25.9 (20.6–32.3)	0.13
	yes	73.8 (45.1–91.7)		27.7 (23–35.7)	
Exogenous hormones	no	68.2 (45.7–89.6)	0.35	26.1 (21.3–33.4)	0.66
	yes	61.9 (49.6–82.1)		25.8 (21–30.7)	
Smoking	no	67 (49.6–85)	0.72	26.6 (21.7–34)	0.12
	yes	65.5 (45.7–86.9)		24.1 (20.6–31)	
Alcohol	no	63.8 (43.5–84.7)	0.11	26.3 (21.4–33.4)	0.77
	yes	69.3 (52.8–91.7)		26 (21–31.6)	
Breast pain	no	61.7 (42.2–83.5)	0.02	25.6 (21.3–32.3)	0.78
	yes	72.2 (518–90.8)		26.3 (21–33)	
Ever being pregnant	no	72.2 (52.8–93.6)	0.001	26.8 (22.03–35.1)	0.07
	yes	61.7 (42.2–80.03)		25.1 (20.9–31.2)	

BC—breast cancer, IQR—interquartile range; * Mann–Whitney *U* test.

## References

[1] Macias H., Hinck L. (2012). Mammary gland development. Wiley Interdiscip. Rev. Dev. Biol..

[2] Pandya S., Moore R.G. (2011). Breast Development and Anatomy. Clin. Obstet. Gynecol..

[3] Arendt L.M., Kuperwasser C. (2015). Form and function: How estrogen and progesterone regulate the mammary epithelial hierarchy. J. Mammary Gland. Biol. Neoplasia.

[4] Ketcham S.A., Sindelar W.F. (1975). Risk factors in breast cancer. Prog. Clin. Cancer.

[5] Gefen A., Dilmoney B. (2007). Mechanics of the normal woman’s breast. Technol. Health Care.

[6] Palmeri M.L., Nightingale K.R. (2011). What challenges must be overcome before ultrasound elasticity imaging is ready for the clinic?. Imaging Med..

[7] Tanter M., Bercoff J., Athanasiou A., Deffieux T., Gennisson J.-L., Montaldo G., Muller M., Tardivon A., Fink M. (2008). Quantitative Assessment of Breast Lesion Viscoelasticity: Initial Clinical Results Using Supersonic Shear Imaging. Ultrasound Med. Biol..

[8] Barr R.G. (2010). Real-Time Ultrasound Elasticity of the Breast. Ultrasound Q..

[9] Barr R.G., Destounis S., Lackey L.B., Svensson W.E., Balleyguier C., Smith C. (2012). Evaluation of breast lesions using sonographic elasticity imaging: A multicenter trial. J. Ultrasound Med..

[10] Berg W.A., Cosgrove D., Doré C.J., Schäfer F.K.W., Svensson W.E., Hooley R.J., Ohlinger R., Mendelson E.B., Balu-Maestro C., Locatelli M. (2012). Shear-wave Elastography Improves the Specificity of Breast US: The BE1 Multinational Study of 939 Masses. Radiology.

[11] Bojanic K., Katavic N., Smolic M., Peric M., Kralik K., Sikora M., Vidačić K., Pacovski M., Štimac D., Ivanac G. (2017). Implementation of Elastography Score and Strain Ratio in Combination with B-Mode Ultrasound Avoids Unnecessary Biopsies of Breast Lesions. Ultrasound Med. Biol..

[12] Dominković M.D., Ivanac G., Kelava T., Brkljacic B. (2015). Elastographic features of triple negative breast cancers. Eur. Radiol..

[13] Rzymski P., Skórzewska A., Skibińska-Zielińska M., Opala T. (2011). Factors influencing breast elasticity measured by the ultrasound Shear Wave elastography–preliminary results. Arch. Med Sci..

[14] Athanasiou A., Tardivon A., Tanter M., Sigal-Zafrani B., Bercoff J., Deffieux T., Gennisson J.-L., Fink M., Neuenschwander S. (2010). Breast Lesions: Quantitative Elastography with Supersonic Shear Imaging—Preliminary Results. Radiology.

[15] Tešić V., Kolarić B., Znaor A., Kuna S.K., Brkljacic B. (2012). Mammographic Density and Estimation of Breast Cancer Risk in Intermediate Risk Population. Breast J..

[16] Rzymski P., Skórzewska A., Opala T. (2011). Changes in ultrasound shear wave elastography properties of normal breast during menstrual cycle. Clin. Exp. Obstet. Gynecol..

[17] Rzymski P., Wysocki P., Kycler W., Opala T. (2011). Correlation between insulin resistance and breast elasticity heterogeneity measured by shear wave elastography in premenopausal women—A pilot study. Arch. Med Sci..

[18] Barr R.G., Zhang Z. (2012). Effects of precompression on elasticity imaging of the breast: Development of a clinically useful semiquantitative method of precompression assessment. J. Ultrasound Med..

[19] Krouskop T.A., Wheeler T.M., Kallel F., Garra B.S., Hall T.J. (1998). Elastic moduli of breast and prostate tissues under compression. Ultrason. Imaging.

[20] Sinkus R., Tanter M., Xydeas T., Catheline S., Bercoff J., Fink M. (2005). Viscoelastic shear properties of in vivo breast lesions measured by MR elastography. Magn. Reson. Imaging.

[21] McKnight A.L., Kugel J.L., Rossman P.J., Manduca A., Hartmann L.C., Ehman R.L. (2002). MR Elastography of Breast Cancer: Preliminary Results. Am. J. Roentgenol..

[22] Ellsworth D.L., Ellsworth R.E., Love B., Deyarmin B., Lubert S.M., Mittal V., Hooke J.A., Shriver C.D. (2004). Outer Breast Quadrants Demonstrate Increased Levels of Genomic Instability. Ann. Surg. Oncol..

[23] Sarp S., Fioretta G., Verkooijen H.M., Vlastos G., Rapiti E., Schubert H., Sappino A.-P., Bouchardy C. (2006). Tumor Location of the Lower-Inner Quadrant Is Associated with an Impaired Survival for Women With Early-Stage Breast Cancer. Ann. Surg. Oncol..

[24] Hallberg Ö., Johansson O. (2010). Sleep on the right side—Get cancer on the left?. Pathophysiology.

[25] Engstrom J.L., Meier P.P., Jegier B., Motykowski J.E., Zuleger J.L. (2007). Comparison of Milk Output from the Right and Left Breasts During Simultaneous Pumping in Mothers of Very Low Birthweight Infants. Breastfeed. Med..

[26] Amarosa A.R., McKellop J., Leite A.P.K., Moccaldi M., Clendenen T.V., Babb J.S., Zeleniuch-Jacquotte A., Moy L., Kim S. (2013). Evaluation of the kinetic properties of background parenchymal enhancement throughout the phases of the menstrual cycle. Radiology.

[27] Chen J.H., Chen W.P., Chan S., Yeh D.C., Su M.Y., McLaren C.E. (2013). Correlation of endogenous hormonal levels, fibroglandular tissue volume and percent density measured using 3D MRI during one menstrual cycle. Ann. Oncol..

[28] Hovhannisyan G., Chow L., Schlosser A., Yaffe M.J., Boyd N., Martin L.J. (2009). Differences in Measured Mammographic Density in the Menstrual Cycle. Cancer Epidemiol. Biomark. Prev..

[29] Buist D.S., Bowles E.J.A., Miglioretti D.L., White E. (2006). Mammographic Breast Density, Dense Area, and Breast Area Differences by Phase in the Menstrual Cycle. Cancer Epidemiol. Biomark. Prev..

[30] Chan S., Su M.-Y.L., Lei F.-J., Wu J.-P., Lin M., Nalcioglu O., Feig S.A., Chen J.H. (2011). Menstrual cycle-related fluctuations in breast density measured by using three-dimensional MR imaging. Radiology.

[31] Wojcinski S., Cassel M., Farrokh A., Soliman A.A., Hille U., Schmidt W., Degenhardt F., Hillemanns P. (2012). Variations in the Elasticity of Breast Tissue During the Menstrual Cycle Determined by Real-time Sonoelastography. J. Ultrasound Med..

